# Case of Diffused Traumatic Femoral Aneurism

**Published:** 1864-03

**Authors:** J. G. Dudley

**Affiliations:** P. A. C. S., in charge of First Division, Winder Hospital.


ART. II.—*Case of Diffused Traumatic Femoral Aneurism.* Reported by Surgeon J. G. DUDLEY, P. A. C. S., in charge of First Division, Winder Hospital.

Private J. A. Coble, company "F," 45th North Carolina Volunteers—aged about twenty years, and a farmer by occupation previous to enlistment—was transferred from the fourth division of Winder Hospital to the first division on the 21st of January, 1864. While in the fourth division, the patient was under the immediate care of Assistant Surgeon Meek, who has kindly furnished the following history of the case:

"J. A. Coble, a private in company "F," 45th North Carolina, was wounded by a Minie ball November 27th, 1863. The ball entered the left thigh, upper third, anteriorly, and coursing inwards and downwards, made its exit at middle third, posterior surface. Said soldier entered the hospital December 2d. The wound healed rapidly without any suppuration or any unpleasant symptoms, and was completely closed by the 8th of December. He was sent before the board on the 13th instant, and a furlough granted. Before the return of the furlough, however, he was suddenly seized, on the evening of the 16th instant, with very severe pain and swelling of the part. I was called to him and administered morphia in such doses as to relieve pain. Afterwards, compression and anodyne poultices were used; under which treatment the pain entirely subsided, as did also the swelling to a great extent. On the 23d December, there was again sudden increase of swelling, with severe pain. Under similar treatment, the pain was again relieved, and the swelling partially subsided. On the 8th of January, the swelling returned the third time, and remained until his transfer to the first division, January 21st, 1864. Prior to the 20th of January, the patient was free from fever."

*Condition of Patient when admitted, January 21st, in the First Division.*

I found a very large and firm swelling over the anterior and lateral portions of the left thigh, superior part; the largest circumference of which was near the junction of the upper and middle thirds of limb. There was but little discoloration of the integuments; pulsation of the common femoral artery very strong; but no pulsation could be detected in the popliteal, anterior or posterior tibial arteries. Patient complained of pain all the while, but at times it was very severe. On auscultation, a faint, yet distinct, *bruit* could be detected. Compression of the common femoral artery caused a cessation of the sound, but did not sensibly diminish the size of the tumor; the leg and foot were cooler than natural, but there was œdema of these parts. Along with these local symptoms there was considerable febrile movement. There were anorexia, slightly furred tongue, constipation, emaciation and debility. Warm laudanum fomentations to the swollen part and the occasional exhibition of quarter grain of sulphate of morphia gave temporary relief. Thus the case progressed from "bad to worse," until it seemed that, unless soon relieved, the patient would die from the sudden bursting of the tumor. On consultation with Professor Gibson and Surgeons Read and Chambliss, a case of "diffused traumatic femoral aneurism" was clearly made out, and an operation at once decided on, which was performed, as follows:

The object in view was to "cut down freely in the tumor, find the orifice of the wounded artery, and cast a ligature above and below it." The common femoral being perfectly compressed by Professor Gibson, an incision of ten inches in length was made, commencing about one inch below Poupart's ligament, and carried down, to the course of the Sartorius muscle, to the lowest boundary of the tumor. A large mass of clotted blood was promptly turned out, and the femoral vessels easily found. The artery was wounded about four inches below Poupart's ligament. Two ligatures, one on the proximal and the other on the distal end of the wounded artery, were applied. It was now ascertained that the femoral vein was likewise wounded, necessitating the application of a ligature to that vessel. In the mass of coagulated blood was found a large cup of fibrine. This incision, though so extensive, did not cause the reduction of the swelling. There still remaining a considerable swelling on the outer and lateral aspect of the limb, a counter opening was made, about five inches in length, which led to another large depot of coagulated blood. This was turned out, as in the first case. At the bottom and upper part of this cavity was found and removed a beautiful cup of almost perfectly pure laminated fibrine, which had been developed on the outer aspect of the artery, and was doubtless partially detached from the vessel This cup had evidently been formed some time, from the fact of its coloring matter having been almost entirely absorbed. Dimensions: the first cup was irregularly ovoid, about half the size of a large hen egg; the second more perfect and about the size of a hickory nut, hollowed out and about half an inch in thickness; the cavities were found to communicate by a small opening opposite the wounded vessels. They were now thoroughly cleansed by means of a large syringe and warm water. At the suggestion of Dr. Read, the ligatures were brought out through the opening connecting the two cavities, and secured outside the counter-opening, in order the more certainly to affect union of the large incision by the first intention, and also to admit of free drainage. The lips of the wound were now brought together and secured by a number of interrupted sutures, supported by adhesive strips. Over this was laid a light compress, secured by Scultetus' bandage. The leg was enveloped, as high as the knee, with cotton batting, and a roller bandage applied from the toes to the knee, and over this a broad flannel roller. The limb was placed in an easy position, and quarter of a grain of sulphate of morphia administered. In about twenty-four hours, the circulation was pretty well re-established in the limb; patient experiencing no pain or inconvenience from the operation. For the first few days, there were fever and considerable constitutional disturbance, with constipation of the bowels. An ounce of castor oil was administered, which procured relief. The fever continuing, I gave the following, which I continued for several days: Sixteen pills of sulphate of quinine, one grain each; one every three hours. Also, tincture of chloride of iron, twenty drops three times a day. At night, quarter grain of sulphate of morphia was given to procure rest. The diet has been light and nutritious. On the fourth day, the wound was examined and found to have united almost by first intention. Patient has steadily improved; gaining strength and spirits daily; not a single untoward symptom has occurred, and to-day, (February 9th,) seventeen days after the operation, he is almost entirely well.

February 11th.—Examined wound this morning and find that the external incision has nearly healed; suppuration now slight and perfectly healthy; internal incision entirely healed; the ligatures have not yet come away; no pulsation in popliteal artery; temperature natural; general health improving rapidly.

